# Objectively measured arm use in daily life improves during the first 6 months poststroke: a longitudinal observational cohort study

**DOI:** 10.1186/s12984-021-00847-x

**Published:** 2021-03-19

**Authors:** G. R. H. Regterschot, J. B. J. Bussmann, Malou H. J. Fanchamps, Carel G. M. Meskers, Gerard M. Ribbers, Ruud W. Selles

**Affiliations:** 1 Department of Rehabilitation Medicine, Erasmus University Medical Center Rotterdam, PO Box 2040, 3000 CA Rotterdam, The Netherlands; 2 Department of Rehabilitation Medicine, VU University Medical Center, De Boelelaan, 1117, 1081 HV Amsterdam, The Netherlands; 3 Rijndam Rehabilitation, Westersingel 300, 3015 LJ Rotterdam, The Netherlands; 4 Department of Plastic and Reconstructive Surgery, Erasmus University Medical Center Rotterdam, PO Box 2040, 3000 CA Rotterdam, The Netherlands

**Keywords:** Accelerometry, Sensor, Ambulatory monitoring, Stroke, Upper extremity, Rehabilitation

## Abstract

**Background:**

It is unclear how arm use in daily life changes after stroke since studies investigating the change in arm use poststroke are scarce. The aim of this study was to investigate the change in arm use during the first six months poststroke. Secondary aim was to compare arm use changes between arm recovery clusters.

**Methods:**

Arm use was measured during week 3, 12, and 26 poststroke with accelerometers on the wrists and the nonaffected leg. Outcomes were the amount of affected and nonaffected arm use during sitting and standing per day and per sit/stand hour, and the daily ratio between arms. Arm function was measured with the Fugl-Meyer Upper Extremity Scale to identify recovery clusters (poor/moderate/excellent). Generalized estimating equations compared arm use outcomes between time points and between recovery clusters.

**Results:**

Thirty-three stroke patients participated. Affected arm use per day increased between week 3 and 12 (30 %; p = 0.04) and it increased per sit/stand hour between week 3–12 (31 %; p < 0.001) and between week 3 and 26 (48 %; p = 0.02). Nonaffected arm use per day decreased between week 3 and 12 (13 %; p < 0.001) and between week 3 and 26 (22 %; p < 0.001) and it decreased per sit/stand hour between week 3 and 26 (18 %; p = 0.003). The daily ratio increased between week 3 and 12 (43 %; p < 0.001) and between week 3 and 26 (95 %; p < 0.001). Changes in arm use did not differ significantly between recovery clusters (p = 0.11–0.62). Affected arm use was higher in the excellent recovery cluster (p < 0.001).

**Conclusions:**

Affected arm use and the ratio between arms increase during the first 26 weeks poststroke especially in patients with excellent arm recovery.

**Supplementary Information:**

The online version contains supplementary material available at 10.1186/s12984-021-00847-x.

## Background

Approximately 80 % of all stroke patients experience impairments in arm function in terms of muscle strength, range of motion, coordination, and voluntary control, resulting in difficulty carrying out daily life activities, loss of independence, and reduced participation [1, 2]. Improving arm function by intensive use and exercise of the affected arm is an essential part of stroke rehabilitation [2, 3]. Improvements in arm function are assumed to translate to improvements in arm use in daily life, i.e., the activities a person does with the arm in the daily life environment. However, there is not much evidence for this assumption. Cross-sectional studies indicate a discrepancy between arm function and arm use after stroke by showing that arm function needs to reach a certain threshold level before arm use in daily life starts to increase [4]. Moreover, longitudinal studies investigating the change in arm use after stroke are scarcely available.

So far only two studies investigated the change in arm use poststroke. A study by Doman et al. (2016) in 15 patients with different times poststroke (22–497 days since stroke) suggests that arm use can increase during outpatient rehabilitation since improvements were observed in two patients [5]. Waddell et al. (2019) found in 29 stroke patients that affected arm use increased during the first 12 weeks after stroke from approximately 2.6 h per day in week 2 poststroke to almost 5 h per day in week 12 poststroke [6].

The two aforementioned studies applied wrist-worn accelerometers for the measurement of arm use after stroke. However, a disadvantage of wrist-worn accelerometers is that they record all arm movements as arm use, including whole-body movements (e.g., walking), resulting in an overestimation of arm use. To avoid an effect of whole-body movements, we developed an arm use monitor that measures arm use by recording arm movements only during sitting and standing and not during whole-body movements such as walking. This arm use monitor consists of two wrist-worn accelerometers and an accelerometer on the nonaffected leg to detect body postures and movements. In a previous study, we showed that this system has adequate accuracy for measuring arm use in stroke patients compared with video recordings [7], indicating that it is a valid tool for measuring arm use poststroke.

Currently, it is unclear how arm use changes after stroke since previous research systematically investigated the change in arm use only during the first 12 weeks poststroke and did not correct the arm use measurements for the effect of whole-body movements. Therefore, the main aim of this study was to investigate the change in arm use during the first 26 weeks poststroke by applying an arm use monitor that corrects arm use measurements for the effect of whole-body movements. Since it is unclear whether recovery of arm function is associated with improvements in arm use, the secondary aim of this study was to compare the change in arm use between different arm recovery clusters.

## Methods

### Participants

In the present study, we aimed to include at least 27 participants since this sample size would enable us to detect a medium effect (Cohen’s d = 0.50) in the change in arm use with an alpha of 0.05 and a power of 0.80. We included people entering Rijndam Rehabilitation (Rotterdam, The Netherlands) after an ischemic or hemorrhagic stroke that suffered from a paretic arm or leg (defined as National Institutes of Health Stroke Scale (NIHSS) 5 A/B or 6 A/B 4 ≥ score > 0). They had to be (1) 18 years or older, (2) had a Mini-Mental State Examination (MMSE) > 19, and (3) were able to sit at least 30 min with back support. We excluded patients who were more than 3 weeks poststroke when admitted to Rijndam Rehabilitation. Participants were screened by a researcher between September 2016 and September 2018. All participants gave their written informed consent and the study was approved by the Medical Ethics Committee of Erasmus MC University Medical Center Rotterdam, The Netherlands (MEC-2015-687).

### Procedures

At the start of the study (week 3 poststroke), all participants were inpatient at Rijndam Rehabilitation, where they received usual care for people after stroke. The usual care program for arm rehabilitation at Rijndam Rehabilitation is based on the principles of the Concise Arm and Hand Rehabilitation Approach in Stroke (CARAS) [3, 8]. The amount and the content of the rehabilitation program were not adapted for this study.

A researcher performed arm use and arm function assessments at 3 weeks, 12 weeks, and 26 weeks poststroke. In addition, the same researcher evaluated stroke severity (National Institutes of Health Stroke Scale (NIHSS) [8, 9]) and collected demographic data including age, gender, affected bodyside, dominant bodyside, admission to the rehabilitation clinic in weeks poststroke, discharge from the rehabilitation clinic in weeks poststroke. Due to individual differences in the usual care, some participants were still at the rehabilitation center at week 12, while at week 26 all participants were at home and were visited by the same researcher for the assessments.

### Arm recovery assessments

The Fugl-Meyer Upper Exterimity assessment (FMUE) was used to measure arm impairments (where impairments refers to a loss of body function and structure) [10, 11]. The FMUE consists of nine components examining voluntary movements and the ability to execute arm movements outside of synergies. The score of the FMUE ranges from 0 to 66, with higher scores indicating a better motor function.

To define arm recovery clusters, we used the classification model of Van der Vliet et al. [12]. This recent study found that different arm recovery clusters exist during the first 6 months poststroke - each with a specific recovery profile - and that recovery cluster belonging can be well-predicted early poststroke. In the present study, we used an online available application (https://emcbiostatistics.shinyapps.io/LongitudinalMixtureModelFMUE/) that implements the model developed by Van der Vliet et al. [12] for the prediction of arm recovery after stroke. For each individual patient, we entered the FMUE data available from week 3, 12 and 26 in the application to identify arm recovery cluster belonging. The model identifies arm recovery cluster belonging as poor, moderate, and excellent based on the initial FMUE after stroke and the amount and rate of recovery in FMUE score poststroke.

### Arm use assessments

**Fig. 1:**
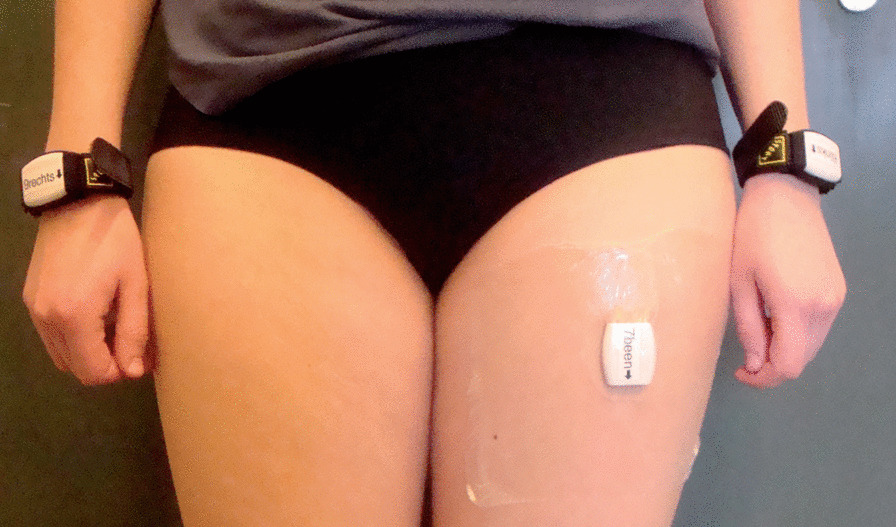
The arm use monitor [7]. The system consists of two wrist-worn accelerometers and an accelerometer on the upper leg. The wrist-worn sensors measure arm movements and the leg sensor determines body postures and movements

We applied an arm use monitor that was developed and validated for the measurement of arm use in stroke survivors [7, 13]. The system is based on the assumption that voluntary arm use is related to arm movement during sitting and standing rather than during whole-body movements such as walking. Arm use is measured by recording arm movement intensity during sitting and standing, thereby avoiding the influence of whole-body movements. The arm use monitor consists of three accelerometers (Activ8 Activity Monitor, Activ8): one attached to the front of the nonaffected thigh to detect body postures/movements (lying/sitting, standing, walking, cycling, running), and one attached to each wrist to measure arm movement intensity (Fig. 1). Each Activ8 accelerometer (30 × 32 × 10 mm, 20 g) measures raw acceleration data with a sample frequency of 12.5 Hz, filters the acceleration data with an exponential moving average filter, and converts these data with a resolution of 1.6 Hz to body postures/movements and movement counts (a commonly used measure of movement intensity) where 1 count is equal to 0.01 g (1 g = 9.81 m/s^2^). The device stores the data in epochs of 30 s—with 48 samples per epoch—and sums the movement counts per epoch. In the present study we examined the number of samples per epoch to ensure that the samples are equal across epochs. A previous study showed that an Activ8 sensor on the upper thigh provides an accurate detection of body postures/movements in stroke survivors (82–100 % accuracy) [13].

Participants were asked to wear the arm use monitor for 1 week (seven days) at three timepoints: week 3, week 12, and week 26 poststroke. The sensors on the wrists were attached with watch-type wristbands and were taken off during the night and during water activities such as showering. The sensor on the nonaffected leg was attached with water-resistant, anti-allergic skin tape, and was worn seven days continuously. During each one week measurement period, the data were stored locally on the sensor devices. After the 1 week measurement, a researcher downloaded the data of the three Activ8 sensors on a PC for data processing and analysis.

All data analysis was performed in R [14] using RStudio (version 1.2.50001, RStudio, Inc.) and a custom-made script based on the study of Fanchamps et al. (2018) that developed and validated the arm use monitor [7]. The first step in the algorithm was to synchronize the Activ8 sensors based on the timestamps within the data files. Then, the measurement period was selected. Only waking hours were analyzed, for which we selected 7 am to 10 pm. Within this period, nonwear of the wrist sensors was detected when at least one device measured zero movement counts for at least one hour. Data were used for analysis when participants had at least two valid days in a measurement week, with a valid day defined as at least ten hours of data without nonwear. In the next step, 30-second epochs were selected in which the posture was sitting or standing according to data of the leg sensor. An epoch was classified as sitting/standing when at least 90 % of the 48 samples were classified as sitting or standing. For each 30-second epoch classified as sitting/standing, arm use was estimated by calculating the total movement counts per wrist-worn sensor. Next, the following arm use outcome measures were calculated per valid day: (1) the total daily movement counts of the affected arm—a measure of the amount of arm movement—during sitting and standing, (2) the total daily movement counts of the nonaffected arm during sitting and standing, (3) the ratio between the total daily use of both arms, calculated as the total daily movement counts of the affected arm during sitting and standing divided by the total daily movement counts of the nonaffected arm during sitting and standing, (4) the mean movement counts of the affected arm per sit/stand hour, and (5) the mean movement counts of the nonaffected arm per sit/stand hour. Finally, per measurement week, a mean daily value was calculated for each arm use outcome measure by averaging across valid days.

### Statistical analysis

All statistical analyses were performed in R [14] using RStudio (version 1.2.50001, RStudio, Inc.). Characteristics of the study participants are described as mean ± SD with minimum and maximum values. Before conducting the statistical analyses, we determined the distribution of the data based on visualizations of the data and normality tests. We used generalized estimating equation (GEE) to investigate how arm use changes over time and to compare the change in arm use between arm recovery clusters. GEE takes into account the dependence between repeated measurements within subjects and can deal with missing data as well as nonnormal distributed data [15]. We developed GEE models for different dependent variables: total daily affected arm use, total daily nonaffected arm use, daily ratio between arms, affected arm use per sitting and standing hour, nonaffected arm use per sitting and standing hour, daily duration of sitting and standing, daily walking duration, daily wearing time of the arm use monitor. To investigate how arm use changes over time, we only included time as factor (three levels: 3, 12 and 26 weeks). To compare the change in arm use between arm recovery clusters, we included time, recovery cluster (two levels: poor/moderate and excellent), and the interaction time ×  recovery cluster as factors. For the development of GEE models, we used the Generalized Estimating Equation package (‘geepack’ package) [16] and set the distribution of the data at ‘gaussian’ and the correlation structure at ‘exchangeable’. A p-value below 0.05 was considered statistically significant. For significant effects in the GEE models, we performed posthoc comparisons with a Bonferroni correction using the Estimated Marginal Means package (‘emmeans’ package) [17]. Change percentages between time points (3, 12 and 26 weeks poststroke) were calculated as: (new value – previous value) / previous value × 100 %.

## Results

**Fig. 2:**
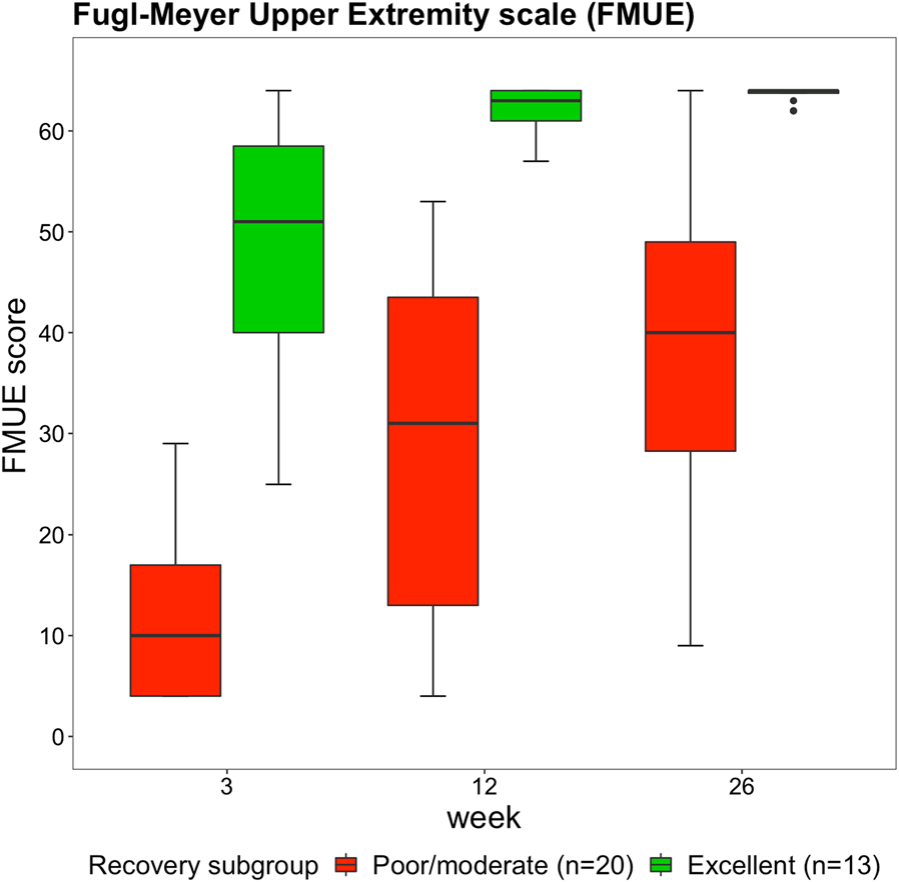
Boxplots showing the Fugl-Meyer Upper Extremity scores. Data are measured at 3, 12 and 26 weeks poststroke in the poor/moderate and excellent arm recovery cluster

In this study 33 stroke patients participated (26 males, seven females). Table 1 presents the characteristics of the patients and Fig. 2 shows the change in FMUE score over time for the different recovery clusters. For the whole sample, FMUE score improved from week 3 to week 12 (p < 0.001), from week 3 to week 26 (p < 0.001), and from week 12 to week 26 (p = 0.008). FMUE scores were higher in the excellent recovery cluster than in the poor/moderate recovery cluster across all time points (p < 0.001). Changes in FMUE score over time were larger in the poor/moderate recovery cluster than in the excellent recovery cluster (p = 0.008).

A complete arm use data set with three measurement weeks (week 3, week 12, week 26) was available from 18 participants. From the other 15 participants, arm use data from two measurement weeks were available. Arm use data were missing at week 3 in three participants: in two participants because of a technical failure of the measurement system, and in one participant no valid measurement days were available due to nonwear. In five participants, arm use data were missing at week 12: in two participants because of a technical failure, in one participant no valid measurement days were available due to nonwear, two participants were not available for the measurements. Arm use data were missing at week 26 in seven participants: in four participants because they dropped out of the study, in two participants because of a technical failure, and in one participant no valid measurement days were available due to nonwear.

**Table 1:** Characteristics of the participants

	Poor/moderate arm function recovery cluster (n = 20)	Excellent arm function recovery cluster (n = 13)
Age in years	57.3 ± 8.5 [39–75]	53.8 ± 10.1 [37–70]
Gender	18 males, Two females	Eight males, Five females
Affected body side	Seven left side, 13 right side	Five left side, Eight right side
Dominant side affected	6 (30 %)	5 (38 %)
Admitted to rehabilitation clinic in weeks poststroke	1.5 ± 0.6 [0.6–3.0]	1.7 ± 0.8 [0.4–3.0]
Discharge from rehabilitation clinic in weeks poststroke	12.4 ± 4.7 [3.9–20.3]	7.5 ± 2.8 [3.7–13.1]
NIHSS^a^ values week 12 poststroke	3.6 ± 2.7 [1–11]	0.08 ± 0.3 [0–1]

Data are reported as mean ± SD [minimal value, maximal value] unless otherwise stated

^a^National Institutes of Health Stroke Scale

**Fig. 3:**
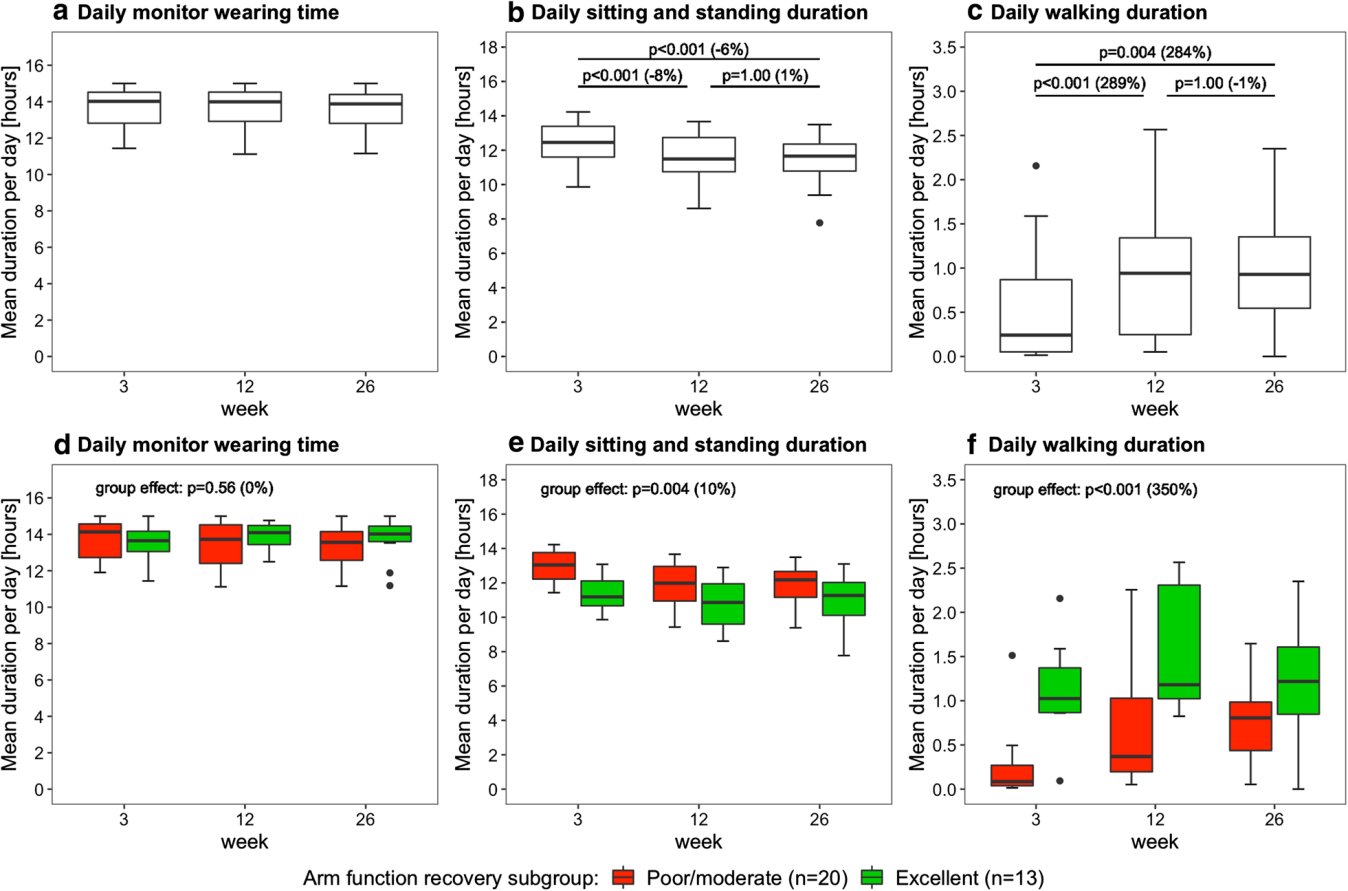
Boxplots showing the daily monitor wearing time, daily sitting/standing duration, and daily walking duration. Data are measured with the arm use monitor in week 3, 12 and 26 poststroke. The percentage between brackets indicates the difference in median value between time points or between clusters. The upper row shows the results for the whole sample (n = 33), the lower row shows the results for the arm recovery clusters

Figure 3 shows the daily monitor wearing time, the daily sitting and standing duration, and the daily walking duration as measured with the arm use monitor. Time poststroke had an effect on daily sitting and standing duration (p < 0.001) and daily walking duration (p < 0.001), but not on daily monitor wearing time (p = 0.73). Posthoc tests revealed that daily sitting and standing duration decreased from week 3 to 12 and from week 3 to 26 (Fig. 3b), and that daily walking duration increased from week 3 to 12 and from week 3 to 26 (Fig. 3c). Furthermore, daily sitting and standing duration was lower in the excellent arm recovery group than in the poor/moderate arm recovery group (Fig. 3e), and daily walking duration was higher in the excellent arm recovery group than in the poor/moderate arm recovery group (Fig. 3f). No time × group interaction effect was found for daily sitting/standing duration (p = 0.33), daily walking duration (p = 0.07), and daily monitor wearing time (p = 0.56), indicating similar changes in both groups over time.

**Fig. 4:**
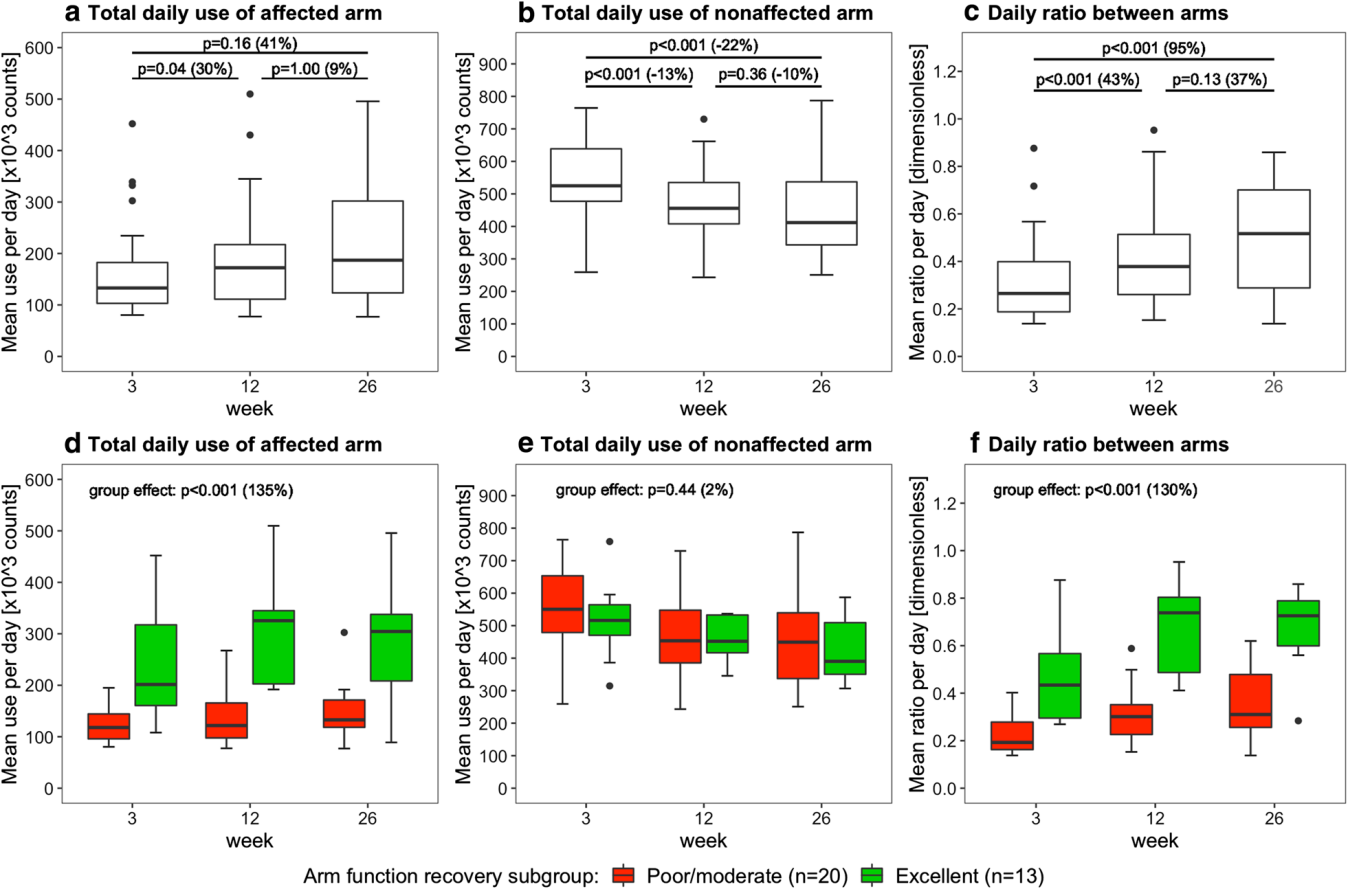
Boxplots showing the daily arm use at week 3, 12 and 26 poststroke. Data are measured with the arm use monitor. The percentage between brackets indicates the difference in median value between time points or between clusters. The upper row shows the results for the whole sample (n = 33), the lower row shows the results for the different arm recovery clusters

**Fig. 5:**
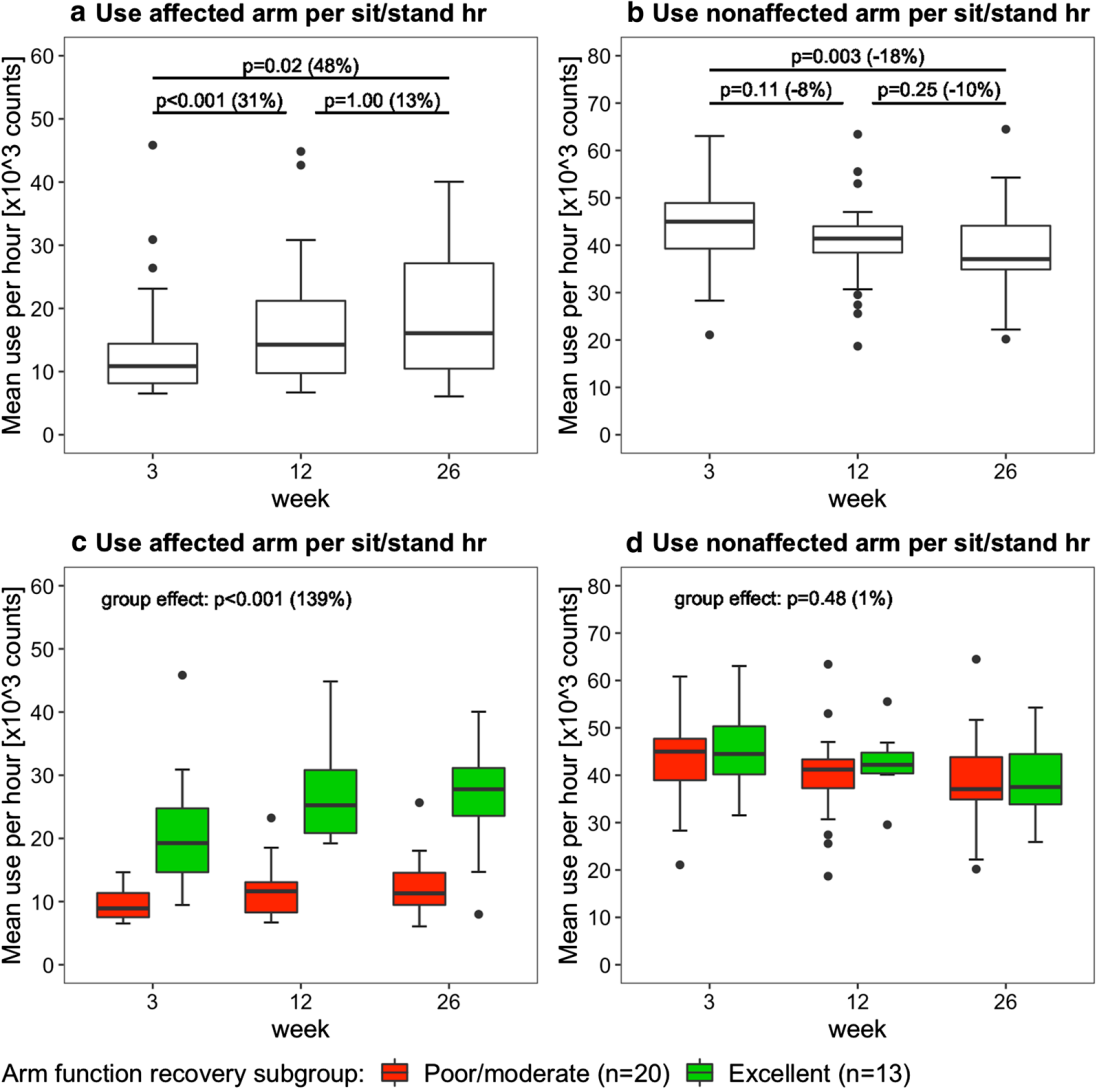
Boxplots showing the arm use per sitting and standing hour. Data are measured with the arm use monitor at week 3, 12 and 26 poststroke. The percentage between brackets indicates the difference in median value between time points or between clusters. The upper row shows the results for the whole sample (n = 33), the lower row shows the results for the different arm recovery clusters

**Fig. 6:**
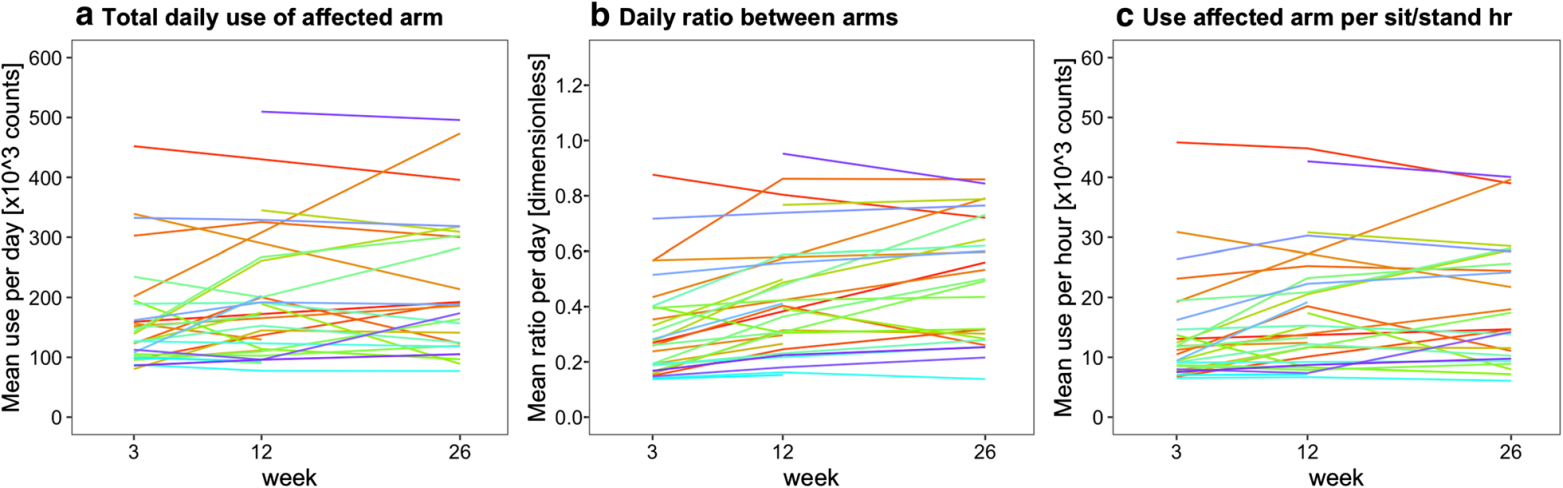
Individual changes in arm use are shown for all patients. Each line represents one patient

Figures 4, 5, and 6 present the arm use outcomes. Time poststroke had an effect on the total daily use of the affected arm (p = 0.04), total daily use of the nonaffected arm (p < 0.001), and the daily ratio between arms (p < 0.001). Posthoc tests revealed that total daily use of the affected arm increased from week 3 to 12, that total daily use of the nonaffected arm decreased from week 3 to 12 and from week 3 to 26, and that daily ratio increased from week 3 to 12 and from week 3 to 26 (Fig. 4a–c). In addition, total daily use of the affected arm and the daily ratio were higher in the excellent arm recovery group than in the poor/moderate arm recovery group (Fig. 4d, f). No time × group interaction effect was observed for total daily use of the affected arm (p = 0.26), total daily use of the nonaffected arm (p = 0.62), and daily ratio (p = 0.25), indicating similar changes in both groups over time.

Since the daily sitting and standing duration decreased over time (Fig. 3B), we corrected arm use outcomes by calculating arm use per sitting and standing hour (Fig. 5). Time poststroke had an effect on affected arm use per sit/stand hour (p < 0.001) and on nonaffected arm use per sit/stand hour (p = 0.004). Posthoc tests showed that affected arm use per sit/stand hour increased from week 3 to 12 and from week 3 to 26 (Fig. 5A), and that nonaffected arm use per sit/stand hour decreased from week 3 to 26 (Fig. 5b). In addition, affected arm use per sit/stand hour was higher in the excellent arm recovery group than in the poor/moderate arm recovery cluster (Fig. 5c). No time × group interaction effect was observed for affected arm use per sit/stand hour (p = 0.11) and nonaffected arm use per sit/stand hour (p = 0.54), meaning that changes were similar in both groups.

## Discussion

This study investigated the change in arm use during the first 26 weeks poststroke by applying an arm use monitor that corrects arm use measurements for the effect of whole-body movements. Results showed increased total daily use of the affected arm, increased affected arm use per sit/stand hour and an improved daily ratio between arms after stroke, especially in patients with excellent arm function recovery. Furthermore, the total daily use of the affected arm, the use of the affected arm per sit/stand hour, and the daily ratio between arms were significantly higher across all time points in the excellent recovery cluster than in the poor/moderate recovery cluster. These findings indicate that recovery of arm function translates to increased use of the affected arm in daily life. The total daily use of the nonaffected arm and the nonaffected arm use per sit/stand hour decreased poststroke.

While the affected arm use per sit/stand hour improved between week 3–12 and week 3–26, the total daily use of the affected arm (defined as total daily affected arm movements during sitting and standing periods) improved only between week 3 and 12 and not between week 3 and 26 since the daily sitting and standing duration decreased poststroke (Fig. 3b). The decrease in daily sitting and standing duration may be the result of a general increase in physical activity during the first 6 months poststroke since we also found an increase in daily walking duration in this period (Fig. 3c). The relatively high nonaffected arm use levels at 3 weeks poststroke may be part of a compensation strategy for the impaired function and limited use of the affected arm. While the nonaffected arm use per sit/stand hour only decreased between week 3 and 26, the total daily use of the nonaffected arm decreased between week 3–12 and week 3–26 since it was affected by the decrease in daily sitting and standing duration over time.

We did not find significant differences in arm use changes between arm recovery clusters. However, this may be due to the small sample sizes of the clusters. Absolute improvements in total daily affected arm use, affected arm use per sit/stand hour, and the daily ratio between arms seem larger in the excellent recovery cluster. For example, the median daily ratio between arms improved from 0.43 (week 3) to 0.73 (week 26) in the excellent recovery cluster, and only from 0.19 (week 3) to 0.31 (week 26) in the poor/moderate recovery cluster (Fig. 4c). The daily ratio between arms in the excellent recovery cluster approaches values in healthy adults (ratio is 0.95 in healthy adults) [18].

Significant differences between recovery clusters were observed in the level of arm use. Patients with excellent arm recovery showed a much higher total daily use of the affected arm, higher daily ratio, and higher use per sit/stand hour of the affected arm than patients with poor/moderate arm recovery. These findings underscore earlier studies that demonstrated that arm use is associated with arm function in stroke survivors [4], and that affected arm use differs between arm function levels [19].

An earlier study by Waddell et al. (2019) found that the mean duration of affected arm use per day increased with almost 100 % from about 2.6 h in week 2 poststroke to almost 5 h in week 12 poststroke [6]. Our results showed a much smaller improvement; the mean affected arm use per day and the mean affected arm use per sit/stand hour improved with respectively 19 % and 27 % from week 3 to 12 after stroke. The smaller improvements in our study may be explained by differences in the rehabilitation program and the difference in the timing of the first assessment (week 2 versus week 3 poststroke) since changes in the first weeks may occur rapidly. Also differences in the sensor-based measurement methods may explain the smaller improvements in our study compared to the study of Waddell et al. (2019). In the present study we measure arm use by recording arm movements only during sitting and standing and not during whole-body movements [7]. The other study measures arm use by recording all arm movements, including whole-body movements such as walking. This results in an overestimation of arm use and arm use recovery since daily walking duration increases significantly after stroke (Fig. 3c), which may explain the larger arm use increases found by Waddell et al. (2019) compared to our study. We are currently investigating the difference between both sensor-based measurement methods by directly comparing the arm use outcomes.

This study has several clinical implications. First, results indicate that the main improvements in arm use occur during the first 12 weeks poststroke. This finding is in line with studies that found that arm function recovery rate is highest during the first three months after stroke [20]. Second, this study shows that arm use improvements gained during the first 12 weeks poststroke are largely retained after 6 months. Third, affected arm use levels were higher and absolute changes in affected arm use seem larger in the excellent recovery cluster than in the poor/moderate recovery cluster. These findings indicate that strategies are needed to support affected arm use in patients with poor/moderate arm recovery. Potential solutions may include arm-hand robotics [21] or wrist-worn sensor-based systems that apply objective feedback to remind and motivate patients to use the affected arm independently outside supervised therapy sessions [22].

A strength of our study compared to previous research is the use of a validated arm use monitor for the measurement of arm use poststroke [7]. The arm use monitor corrects arm use outcomes for the effect of whole-body movements, thereby avoiding that walking influences the arm use outcomes. The present study has several limitations. First, the small sample size and single recruitment site may limit the generalizability of our findings. Second, a complete data set with three measurement weeks were available from only 18 patients. In an additional analysis we compared the reported arm use outcomes of the 33 patients to the arm use outcomes of the complete cases (n = 18) and found that the outcomes are very similar [see Additional file 1]. Hence, the missing data does not have a significant influence on the study outcomes. Third, we did not distinguish between arm use in daily life and arm use in therapy sessions. This might be interesting, since arm use intensity during therapy sessions may be higher and have a relatively strong effect on the arm use measurements. Fourth, the recovery clusters were relatively small which may have prevented us from finding significant differences in arm use changes between recovery clusters. Another limitation is that with our sensor-based method it was not possible to accurately determine the arm use hours per day, since the system determined the arm activity counts per 30 s epoch and not on a more fine-grained scale. This prevented us from comparing the daily hours of arm use with other studies.

## Conclusions

This study shows that the use of the affected arm and the ratio between arms increase during the first 26 weeks after stroke, especially in patients with excellent arm function recovery. Our results indicate that recovery of arm function translates to increased arm use in daily life. Further research with adequate sample sizes of the recovery clusters is required to confirm this. The present study contributes to a better understanding of the change in arm use poststroke and its relationship with arm function recovery, which is essential for optimizing arm use in daily life after stroke.

## Supplementary Information

**Additional File 1** An analysis of the effect of missing data on the study results.

## Data Availability

The datasets used and/or analysed during the current study are available from the corresponding author on reasonable request.

## Abbreviations

CARAS: Concise Arm and Hand Rehabilitation Approach in Stroke
FMUE: Fugl-Meyer Upper Exterimity assessment
GEE: Generalized estimating equation
MMSE: Mini-Mental State Examination
NIHSS: National Institutes of Health Stroke Scale
