# Supplementary material for: Objectively measured arm use in daily life improves during the first 6 months poststroke: a longitudinal observational cohort study
Source: J Neuroeng Rehabil. 2021 Mar 19;18:51. doi: 10.1186/s12984-021-00847-x (PMC7980644; doi:10.1186/s12984-021-00847-x)

Additional file 1

An analysis of the effect of missing data on the study results

To determine the effect of missing data on the study results, we compared the results between two samples:

1) 18 subjects with complete dataset and 15 subjects with 1 missing week.
    - Poor/moderate arm recovery cluster: 20 subjects
    - Excellent arm recovery cluster: 13 subjects

2) 18 subjects with complete dataset.
    - Poor/moderate arm recovery cluster: 12 subjects
    - Excellent arm recovery cluster: 6 subjects

1. Total arm use per day

1) 18 subjects with complete dataset and 15 subjects with 1 missing week.

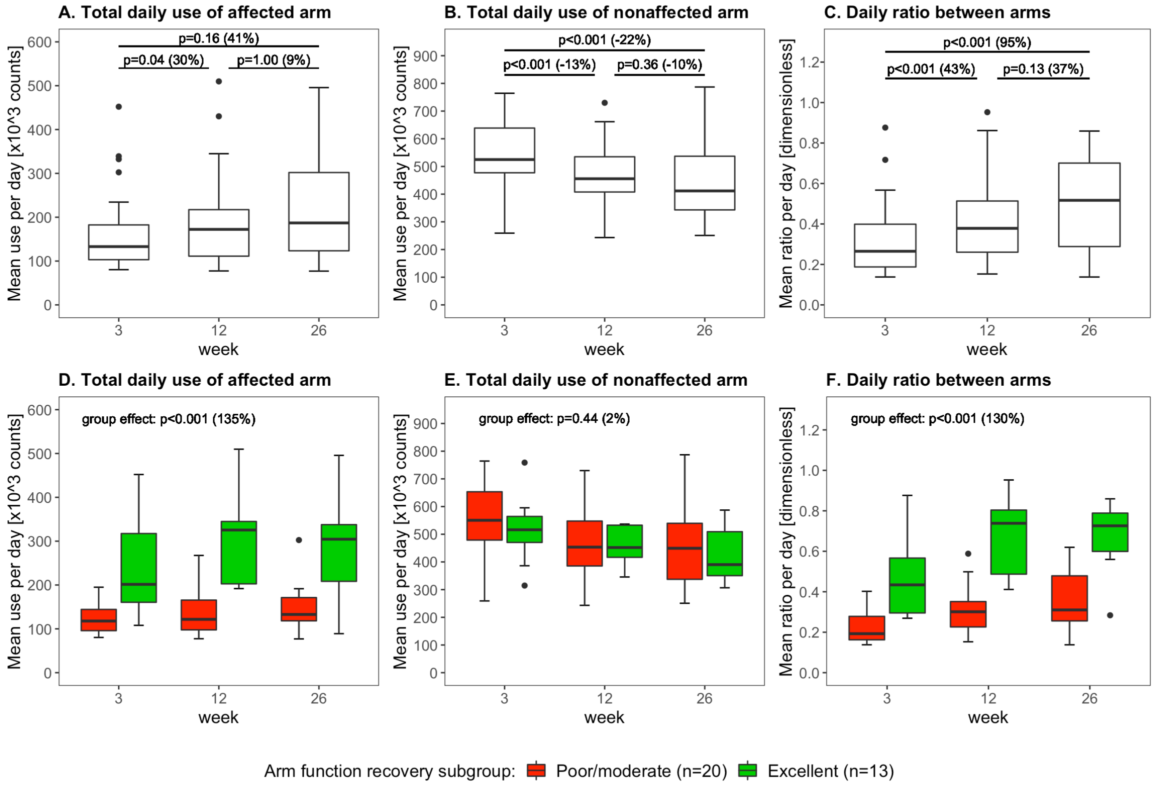

2) 18 subjects with complete dataset.

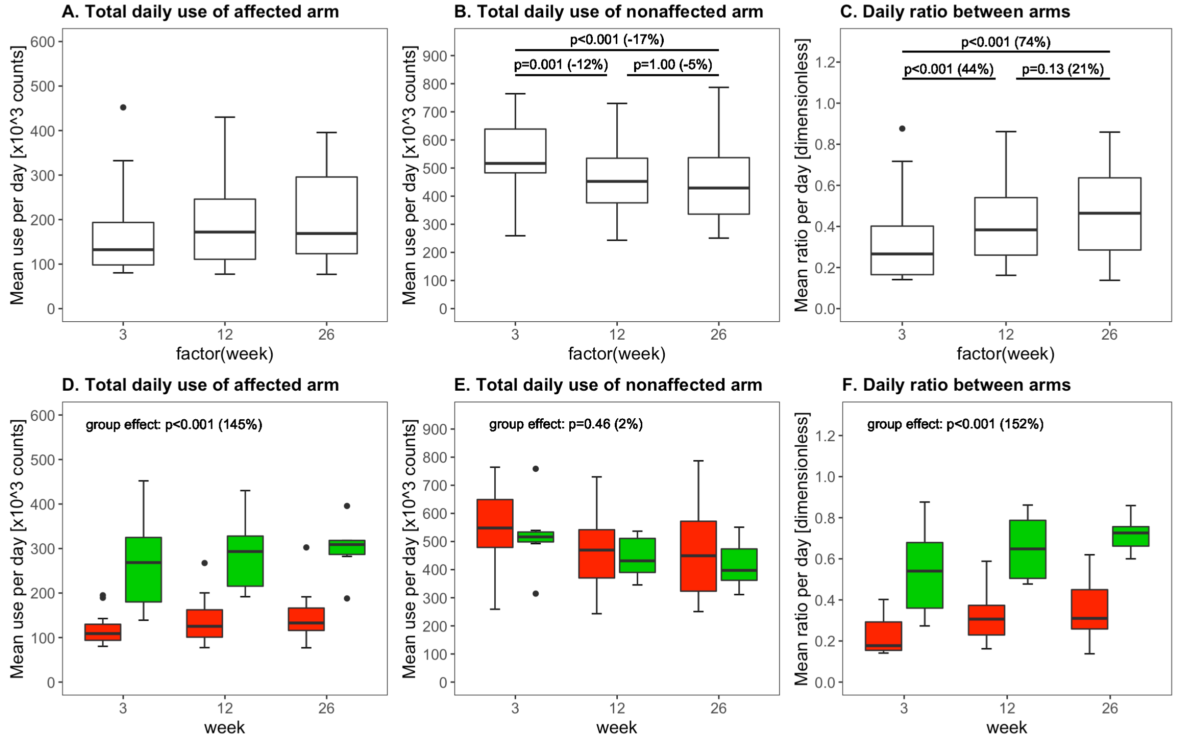

2. Arm use per sit/stand hour

1) 18 subjects with complete dataset and 15 subjects with 1 missing week.

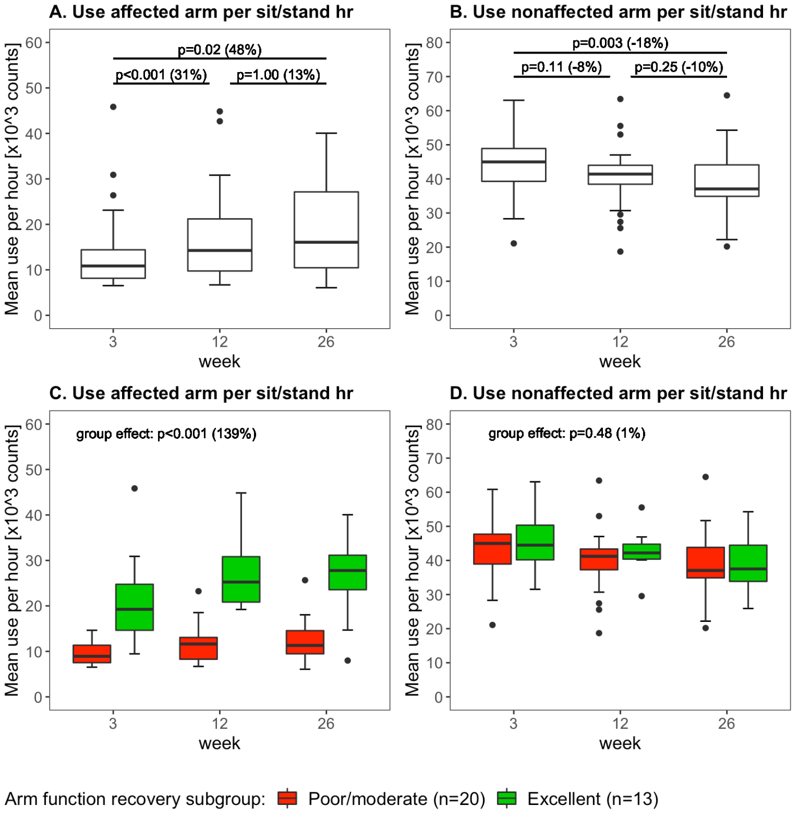

2) 18 subjects with complete dataset.

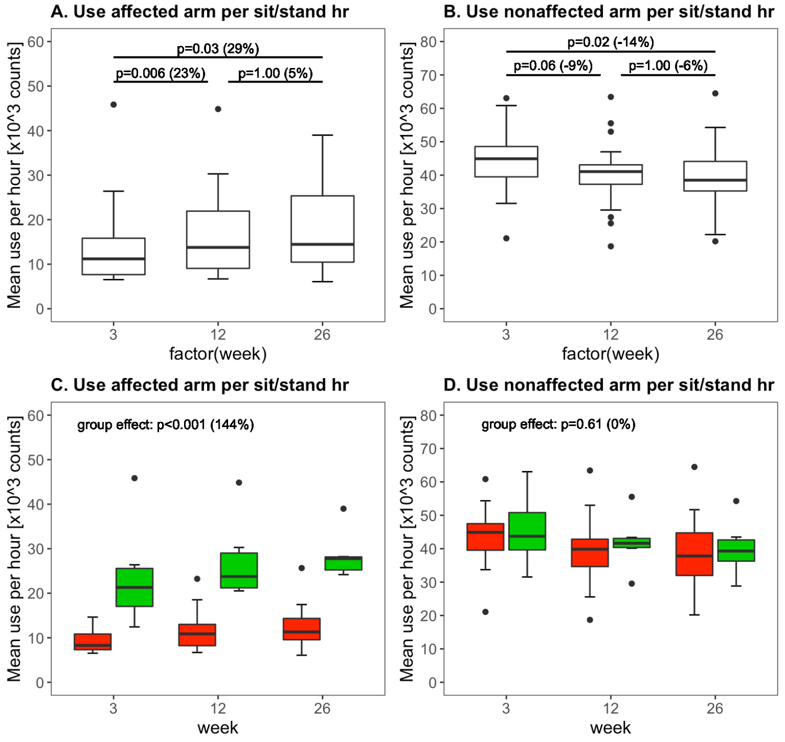

Supplement: Supplementary file 1 — Additional File 1 An analysis of the effect of missing data on the study results. [file 12984_2021_847_MOESM1_ESM.pdf]
